# P-TEFb as A Promising Therapeutic Target

**DOI:** 10.3390/molecules25040838

**Published:** 2020-02-14

**Authors:** Koh Fujinaga

**Affiliations:** Department of Medicine, University of California, San Francisco, CA 94143-0703, USA; koh.fujinaga@ucsf.edu; Tel.: +1-415-502-1908

**Keywords:** P-TEFb, transcription elongation, HIV, cancer, cardiac hypertrophy, RNA polymerase II, infectious diseases, inflammation, autoimmune diseases, inhibitors

## Abstract

The positive transcription elongation factor b (P-TEFb) was first identified as a general factor that stimulates transcription elongation by RNA polymerase II (RNAPII), but soon afterwards it turned out to be an essential cellular co-factor of human immunodeficiency virus (HIV) transcription mediated by viral Tat proteins. Studies on the mechanisms of Tat-dependent HIV transcription have led to radical advances in our knowledge regarding the mechanism of eukaryotic transcription, including the discoveries that P-TEFb-mediated elongation control of cellular transcription is a main regulatory step of gene expression in eukaryotes, and deregulation of P-TEFb activity plays critical roles in many human diseases and conditions in addition to HIV/AIDS. P-TEFb is now recognized as an attractive and promising therapeutic target for inflammation/autoimmune diseases, cardiac hypertrophy, cancer, infectious diseases, etc. In this review article, I will summarize our knowledge about basic P-TEFb functions, the regulatory mechanism of P-TEFb-dependent transcription, P-TEFb’s involvement in biological processes and diseases, and current approaches to manipulating P-TEFb functions for the treatment of these diseases.

## 1. Introduction

Eukaryotic transcription by RNA polymerase II (RNAPII) is a highly orchestrated process regulated at multiple steps, including initiation, promoter clearance, elongation, co-transcriptional processing of nascent transcripts, termination, mRNA cleavage and polyadenylation [1,2,3,4]. Rapid development of genome-wide transcriptome analysis has revealed that elongation is a critical regulatory step of transcription [5,6,7,8,9,10]. The positive transcription elongation factor b (P-TEFb) that contains either Cyclins T1 (CycT1), T2a (CycT2a), or T2b (CycT2b) and CDK9 plays a central role in stimulating transcriptional elongation by phosphorylating serine residues at position 2 (Ser-2) of the conserved heptapeptide repeats (YSPTSPS) of the RNAPII C-terminal domain (CTD) and negative transcription elongation factors (N-Tefs) [11,12,13,14,15,16]. Since P-TEFb functions via direct interaction with viral and cellular transcription factors (TFs) involved in various human diseases and conditions, P-TEFb is recognized as an excellent drug target for infectious diseases (such as HIV/AIDS), inflammation/autoimmune diseases, cardiac hypertrophy, and cancer [17,18,19]. Thus an increasing number of CDK9 inhibitors are currently being developed and tested clinically and pre-clinically [20,21,22,23,24]. In this review, I would like to provide an overview of the structure and function of P-TEFb, human disease models where P-TEFb plays a critical role, current attempts to manipulate P-TEFb functions, and potential problems and solutions for development of effective and specific P-TEFb inhibitors.

## 2. Discovery of P-TEFb

In 1992, Marshall and Price observed that partially purified nuclear fractions stimulate the elongation of transcription by RNAPII in a manner that is dependent on the kinase inhibitor 5,6-dichloro-1-beta-D-ribofuranosylbenzimidazole (DRB) [25]. They named this factor the positive transcription elongation factor b (P-TEFb) and subsequently purified it as a heterodimer of about 124kD and 43kD [26]. Before the discovery of P-TEFb, the initiation/promoter clearance step was considered to be a main regulatory step of RNAPII-dependent transcription. One of the few examples of eukaryotic transcription regulated at the elongation step was human immunodeficiency virus type 1 (HIV), on which RNAPII is paused at the 5′-proximal region (at around nucleotide 20-60), producing only short transcripts that form a stem-loop structure called Trans-Activation Response (TAR) element [27]. Viral Tat protein mediates the release of the paused RNAPII and stimulates the elongation of HIV transcription with help from cellular co-factors [28,29,30]. Rice and colleagues demonstrated that Tat interacts with a cellular protein kinase of about 42kD that hyperphosphorylates Ser 2 of RNAPII CTD, and named this factor Tat-associated kinase (TAK) [31]. P-TEFb was brought under the spotlight when TAK was identified to be cyclin-dependent kinase 9 (CDK9), which was purified and cloned several years before by Giordano and colleagues [32], and CDK9 was identified to be the kinase subunit of P-TEFb [33,34]. Soon after this, Jones and colleagues cloned a previously unknown C-type cyclin from human cells as a factor that binds to Tat and TAR and also rescues the defect of Tat-dependent transcription in rodent cells, and named it Cyclin T1 (CycT1), which was subsequently identified to be a cyclin subunit of P-TEFb [35]. CycT1 in mouse cells is highly homologous to human CycT1, but mouse CycT1 does not bind to TAR due to only one amino acid difference: the position 261 is Cys in human and Tyr in mouse [36,37,38,39,40]. Other cyclins (CycT2a and T2b) do not contain the C261 residue, hence do not support HIV Tat transactivation [41,42]. Following these hallmark studies, P-TEFb research was initially undertaken in the field of HIV [43]. P-TEFb was then found to play a critical role in the development of cardiac hypertrophy [44], followed by a series of discoveries that P-TEFb interacts with various transcription factors (TFs) such as the Nuclear Factor kappa-B (NFκB) [45], cMyc [46,47], hormone receptors [48,49,50] and more (see below). These studies indicate P-TEFb’s involvement in inflammatory/autoimmune diseases and cancers [51]. The list of transcription factors that interact with P-TEFb is increasing [52] (Table 1). Discovery of chromatin-associated factors [53,54], general transcription factors [55], or the Super Elongation Complex [56] as P-TEFb-recruitment factors as well as genome-wide analyses including chromatin immunoprecipitation (ChIP)-seq [57,58,59] have revealed that P-TEFb is a main on/off switch to control transcription elongation [12,52,60,61].

## 3. Protein Structure and Function of P-TEFb Subunits

**Figure 1:**
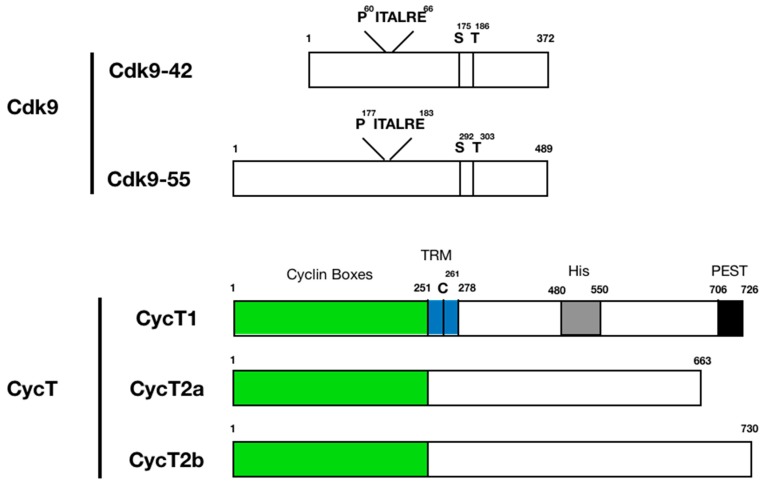
Schematic presentation of Cdk9 and CycT domain structures. There are two isoforms of Cdk9 (Cdk9-42 and Cdk9-55). These two proteins are identical except that CDK9-55 has a longer N-terminal region. Cdk9 contains a unique self-inhibitory sequence (PITALRE) and phosphorylation sites. CycT1, T2a, and T2b (collectively, CycT) contain two highly conservative cyclin box structures in their N-termini, which interact with CDK9 (green). In CycT1, a region immediately after the cyclin boxes (a.a. 251–278) interacts with HIV Tat and TAR via a critical C261 residue, and hence is called the Tat-TAR recognition motif (TRM) (blue). CycT1 contains a region rich in histidine residues (a.a. 480–550) that is required for direct interaction with RNAPII CTD (gray). The C-terminal end (a.a. 706–726) of CycT1 forms a typical PEST motif, which determines its protein stability in cells (black).

CDK9: The *CDK9* gene encodes two isoforms expressed from two alternative transcription start sites in the *CDK*9 gene, CDK9-42 and CDK9-55 [72,73,74] [75] (Figure 1). These two proteins are identical except that CDK9-55 has a longer N-terminal region. In cells, CDK9-42 is the main kinase subunit of P-TEFb. CDK9 is a member of the transcription CDKs (CDKs 7, 8, 9, 10, 11, 12, 13, 19 and 20), which share common structural features of protein kinase domains with cell cycle CDKs. CDK9′s kinase domain forms a two-lobed structure where the catalytic site is sandwiched between N- and C-lobes [51,52,76,77]. The N-lobe forms an inhibitory domain including a helix unique to each CDK (PITALRE for CDK9), and the C-lobe functions as an activation domain that includes a regulatory T-loop structure (Figure 1). CDK9 is activated by phosphorylation of threonine residue (T186) in the T-loop [78,79,80,81,82,83]. Phosphorylation of additional serine residue near the T-loop (S175) also regulates CDK9 activity although the precise molecular mechanism of S175-dependent regulation is still largely unknown [84,85,86] (Figure 1).

CycT: CycT1, T2a, and T2b (collectively, CycT) contain two highly conservative cyclin box structures in their N-termini, which interact with CDK9 [87,88] (Figure 1). A short stretch of basic amino acids immediately after the C-terminal cyclin box serves as a nuclear localization signal. In CycT1, this region (a.a. 251–278) interacts with HIV Tat and TAR via a critical C261 residue, and hence is called the Tat-TAR recognition motif (TRM) [35,36] (Figure 1). The N-terminal 278 amino acids of CycT1 are sufficient for mediating Tat transactivation [36,38,39]. Substitution of amino acids in this region makes potent dominant negative CycT1 mutants that interfere with the activity of endogenous P-TEFb [89,90,91,92]. The C-terminal region is diverse among CycT proteins [93] and, since most structure/function studies have been conducted with CycT1 only, functions of this part in CycT2 are largely undefined. CycT1 contains a region rich in histidine residues (a.a. 480–550) that is required for direct interaction with RNAPII CTD [94] (Figure 1). This region was later identified to promote formation of a phase-separated localization of the P-TEFb complex [95]. The C-terminal end (a.a. 706–726) of CycT1 forms a typical PEST motif, which determines its protein stability in cells [35] (Figure 1).

## 4. Substrates of CDK9

CDK9 was originally cloned as a CDC2-related CDK that phosphorylates the Retinoblastoma (Rb) protein [32,96]. Subsequently, CDK9 was recognized as a transcription elongation factor that stimulates the phosphorylation of RNAPII, by using antibodies that specifically recognize phosphophorylated serines 2 (S2P), or serines 5 (S5P) of the heptapeptide repeat [97,98,99]; [16]. Genome-wide ChIP-seq analyses using these and P-TEFb antibodies have also indicated that the recruitment of P-TEFb coincides with the increase in S2P CTD at the 5′-proximal sites [59,100]. However, two independent studies have demonstrated that S5 phosphorylation by CDK9 is also stimulated by HIV Tat protein [101,102]. Moreover, studies by Geyer and colleagues using a series of purified peptides (3x heptapeptide repeats including phosphorylated amino acids at different sites) have also indicated that CDK9 is a S5 kinase and its phosphorylation can be augmented when the serine 7 (S7) is phosphorylated [103]. In addition, the RD subunit of the negative transcription elongation factor (NELF) is phosphorylated by CDK9 [104,105]. This phosphorylation mark mediates dissociation of NELF from RNAPII [104,105,106]. The Spt5 subunit of DRB sensitivity-inducing factor (DSIF) is also phosphorylated at its CTD-like motif in the C-terminus by CDK9. The phosphorylated DSIF remains associated with RNAPII and becomes a positive transcription factor [107,108,109,110,111]. Thus, CDK9-dependent phosphorylation of these N-TEFs is a checkpoint for the RNAPII pause-release [9,97,112].

Recently, Fisher and colleagues conducted a high throughput search (HTS) for CDK9 substrates and identified ~100 putative substrates, the majority of which are in the categories of transcription and RNA catabolism [113]. Among them is transcription termination factor Xrm2, suggesting that P-TEFb is also involved in the regulation of transcription termination, which is consistent with the observation by Kouzarides and colleagues that aberrant stimulation of P-TEFb activity results in global transcriptional readthrough in ES cells [114]. Interestingly, another HTS search using an analogue-sensitive CDK9 mutant followed by mass spectrometry (MS) conducted by Eick and colleagues identified putative CDK9 substrates that showed a minimum overlap with the putative substrates identified by the Fisher group [113,115]. Further studies are required to complete the list of CDK9 substrates in cells. CDK9 also phosphorylates viral proteins such as Kaposi’s Sarcoma Associate Herpesvirus (KSHV) RTA proteins, which modulate viral replication [116].

## 5. Regulation of P-TEFb Functions in Cells

**Figure 2:**
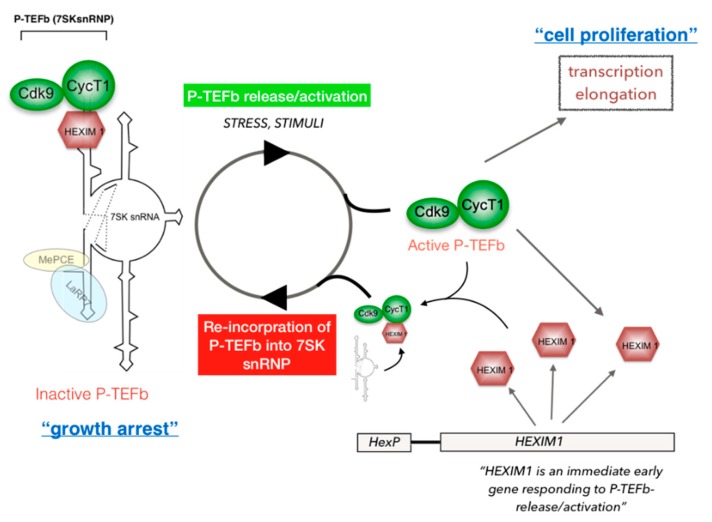
P-TEFb regulatory mechanism. In cells, most P-TEFb molecules are incorporated into 7SK snRNP which contains 7SK snRNA, HEXIM1, MePCE, and LARP7. In 7SK snRNP, the CycT1 subunit directly binds to the central loop of 7SK snRNA and HEXIM1, which inhibits the kinase activity of Cdk9. Various stimuli including stress, environmental stimuli, cytokine signaling, PKC activation, and treatment of cells with HDACis, BETis, and other compounds release P-TEFb and stimulate Cdk9 kinase activities. Released (free) P-TEFb can subsequently be recruited to RNAPII early elongation complex paused at the promoter proximal regions of many cellular genes that drive cell proliferation. One of P-TEFb’s target genes immediately responding to P-TEFb release/activation is its own inhibitor *hexim1*, and newly expressed HEXIM1 by P-TEFb re-incorporates P-TEFb into 7SK snRNP, resulting in cell growth arrest.

In growing cells, P-TEFb partitions between an active complex, alone or associated with various proteins that recruit P-TEFb to its target genes where RNAPII is engaged (free P-TEFb), and an inactive complex with 7SK small nuclear RNA (7SK snRNA), hexamethylene bisacetamide-inducible mRNAs 1 and 2 (HEXIM1/2) proteins, La-related protein 7 (LARP7) and methyl phosphate capping enzyme (MePCE), also known as the 7SK small nuclear ribonucleoprotein (7SK snRNP) [117,118,119,120,121,122,123,124,125] (Figure 2). Depending on cell type, 50% to 90% of P-TEFb is found in the 7SK snRNP. This P-TEFb equilibrium maintains the level of active P-TEFb that stimulates transcription elongation necessary to determine the state of cellular activation, proliferation, and differentiation [14,15] (Figure 2). Dysregulation of the P-TEFb equilibrium, therefore, causes various diseases [19,76]. For instance, disrupting 7SK snRNP in cardiomyocytes results in upregulation of global gene expression, leading to cardiac hypertrophy [44]. Many stresses such as UV light, heat, inhibition of transcription, and specific intracellular signaling cascades release P-TEFb from the 7SK snRNP [15,20,126,127]. Also, small compounds such as histone deacetylase inhibitors (HDACis), which include tricostatin A and suberoylanilide hydroxamic acid (SAHA); PKC agonists; bromodomain and extra-terminal (BET) bromodomain inhibitors (BETis); DNA damage-inducing agents; a strong cell-differentiation inducer, hexamethylene bisacetamide (HMBA); and others also release P-TEFb from 7SK snRNP via various known and unknown mechanisms and activate transcription elongation mediated by P-TEFb [14,15,60,128,129,130,131,132] (Figure 2). After being released from 7SK snRNP, active P-TEFb is recruited to its target genes, many of which are pro-proliferative [126,133,134,135]. However, one of the immediate early genes responding to the P-TEFb-release is the *hexim1* gene, and newly produced HEXIM1 proteins immediately re-incorporate P-TEFb into 7SK snRNP, inducing cell growth arrest (Figure 2) [136,137,138]. This negative feedback mechanism explains why many anti-cancer compounds are found to be very potent P-TEFb-releasers/activators [128,129,132,137,138,139,140].

## 6. Mechanism of P-TEFb Recruitment to Its Target Genes

**Figure 3:**
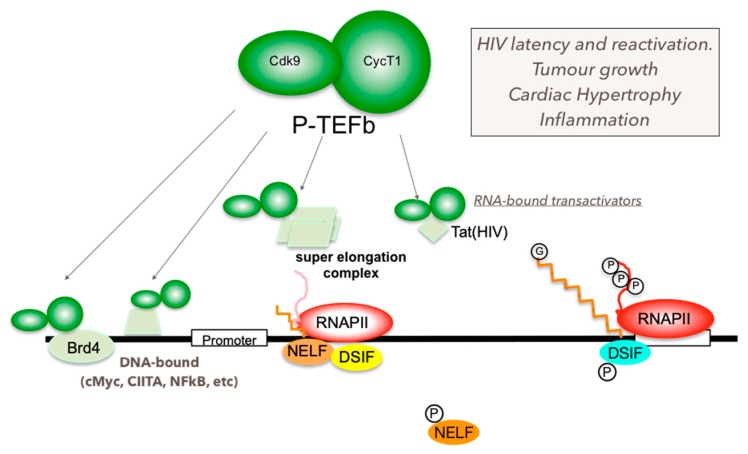
P-TEFb (CycT1:CDK9) is recruited to the RNAPII transcription machinery by various factors such as the epigenetic factor Brd4, DNA-bound transactivators, RNA-bound transactivtors, or Super Elongation Complexes. Aberrant regulation of P-TEFb functions causes various diseases and conditions including HIV latency/reactivation, inflammation, tumor growth and cardiac hypertrophy.

Recent developments in genome-wide studies and bioinformatics analysis have caused a paradigm shift through awareness that transcription of most cellular genes is regulated at the elongation step [58,59,97,100]. RNAPII is found to bind to many promoters but stalls immediately after initiation of transcription mainly due to N-TEFs, NELF and DSIF in the basal (uninduced) state [58,59,97,100] (Figure 3). Recruitment of P-TEFb upon transcriptional cues/stimuli is a key checkpoint of RNAPII pause-release and subsequent induction of transcription elongation [9,97,112]. P-TEFb is recruited to its target genes by various gene-specific or gene-nonspecific binding partners including (a) chromatin-binding proteins such as the Bromodomain-containing protein 4 (Brd4) [53,54], (b) factors directly associated with RNAPII such as PolII-Associated factor 1c (PAF1c) [111,141,142], (c) components of the Mediator complex such as Med26 [55], (d) components of the Super Elongation Complex, such as AFF4 [56,143,144,145], (e) DNA-bound transactivators such as NFκB [45], cMyc [46,47,133], the Signal Transducer and Activator of Transcription (STAT)3 [63,146,147], the Myoblast Determination Protein 1 (MyoD) [62,148], Myocyte Enhancer Factor-2 (MEF2) [65], and hormone receptors including Estrogen Receptor (ER) and Androgen Receptor (AR) [48,49], (f) DNA-unbound transactivators such as the Autoimmune Regulator (AIRE) and the Class II Transactivator (CIITA) [66,67,149], and (g) RNA-bound transactivators such as HIV Tat [150,151,152,153,154,155] (Figure 3) (Table 1). Although many P-TEFb-recruitment factors have been identified, how these factors coordinate with each other and respond to different transcriptional cues (cell signaling, stress, developmental stages, etc.) is largely unknown. An interesting example, which is also important to consider for development and usage of compounds targeting P-TEFb, is a treatment with BET inhibitors (BETi) [156,157,158]. When cells are treated with BETis such as JQ1 and iBET, the Brd4/P-TEFb complex bound to Histone H3 acetylated at the lysine residue 27 (H3K27Ac) is removed from actively transcribing chromatin loci within 30 min, suppressing P-TEFb-dependent transcription of genes associated with transcriptionally active histone modification, H3K27Ac [158]. Simultaneously, BETis release P-TEFb from its inactive pools in 7SK snRNP, and activate P-TEFb-dependent transcription within 1 h [117] (presumably due to cellular stresses by sudden changes in chromatin structure upon removal of Brd4, [159]). This P-TEFb release also triggers HEXIM1 upregulation and subsequent suppression of P-TEFb-dependent transcription by forming 7SK snRNP [136] (Figure 2). Therefore, the overall effects on cellular gene expression by BETis are a mixture of stimulation and suppression of P-TEFb-dependent transcription, which depends on genes, local chromatin structures, cellular status, cell types and time.

## 7. P-TEFb and Human Diseases

P-TEFb plays critical roles in many human diseases and conditions through various different mechanisms [17,21,51,76,77,135]. Therefore, P-TEFb can be a very promising therapeutic target for the treatment of many diseases. However, because of the complexity of the above-mentioned regulatory mechanisms of P-TEFb and the cellular transcriptional network, development of effective therapeutic methods that may potentially target P-TEFb requires a deeper understanding of P-TEFb-dependent transcriptional regulation. The following are some examples of human diseases where P-TEFb is actively involved.

HIV and AIDS: P-TEFb is an essential cellular co-factor for HIV transcription since CycT1 interacts with the viral Tat protein and with the viral promoter via TAR RNA [43,150,154,160]. The HIV long-terminal repeat (LTR) DNA sequence that acts as a promoter and enhancer for HIV transcription also contains NFκB binding sites, and NFκB also requires P-TEFb for its activity via direct interaction [152,161,162]. Therefore, P-TEFb is recruited for both Tat-dependent and Tat-independent (basal) HIV transcription. Many attempts have been made to use CDK9 kinase inhibitors for suppressing HIV infection [163,164,165,166,167,168,169], although at this point none are approved as anti-HIV drugs. Another obstacle of HIV/AIDS is the latent infection of HIV [170,171,172,173,174,175]. HIV latency is established immediately after a new infection by a quick attenuation of viral transcription by various mechanisms such as epigenetic gene silencing, lack of cellular co-factors, transcription interference, etc. [170,171,172,173,174,175,176]. Since latently infected cells do not express HIV proteins, these cells escape the host’s immune surveillance and clearance. Although combinatory antiretroviral therapy (cART) suppresses viral spread, reduces plasma viremia, and restores CD4+ T cell counts, there still remains a reservoir of latently infected cells with fully replication-competent proviruses, which are scattered in lymphoid and certain non-lymphoid tissues throughout the body [177,178]. Termination of treatment and subsequent immune activation of infected cells can cause rapid viral rebound followed by detectable plasma viremia and reduction of CD4+T cell count [179,180]. Resting CD4+ T cells and their memory subsets harboring silent proviruses are generally considered to represent long-lived viral reservoirs [181,182,183,184]. Interestingly, in resting CD4+ T cells, the protein expression of CycT1 is kept at a vanishingly low level via post-transcriptional mechanisms [185,186,187]. Activation of T cell signaling via T cell receptor engagement and downstream PKC activation increases CycT1 protein levels, which is a mandatory step of HIV reactivation from latently infected cells [184,185,187,188]. Therefore, P-TEFb is also an essential factor to maintain HIV latency. There is still an active debate about whether, to eliminate the viral reservoir, latently infected cells should be eliminated by reactivating HIV and subsequently eliciting viral cytotoxic effects and host immune responses (“shock and kill” approach), or kept latent by constantly suppressing viral production from these cells (“block and lock” approach) [171]. However, for either approach, P-TEFb is a key molecule to control viral gene expression.

Cardiac Hypertrophy: One of the first examples of human diseases other than HIV/AIDS where P-TEFb activity plays a critical role is cardiac hypertrophy [189,190,191,192]. Treatment of cardiomyocytes with hypertrophic stimulations such as endothelin-1 (ET1) or mechanical stresses releases P-TEFb from 7SK snRNP and activates P-TEFb [44]. Conversely, experimental disruption of 7SK snRNP by antisense oligonucleotides results in P-TEFb activation and hypertrophic responses [44]. Finally, mice overexpressing CycT1 exhibit cardiac hypertrophy [44]. Siddiqui and colleagues demonstrated that HEXIM1 (also known as Cardiac Lineage Protein-1 or CLP-1) knockout in mice is lethal in late fetal stages due to heart failure, and that bigenic mice overexpressing CycT1 in a HEXIM (+/-) heterozygote background exhibit enhanced susceptibility to cardiac hypertrophy accompanied by elevated Cdk9 activity [193,194]. In a hypertrophic mice model with an over-expression of calcineurin, the interaction between CDK9 and HEXIM1 in cardomyocytes is diminished, indicating constant up-regulation of P-TEFb activity [188]. Another type of transgenic mouse expressing mutant HEXIM1 lacking its C-terminal 49 amino acids created in the Montano laboratory (there, HEXIM1 is called “EDG1”) exhibits myocardial hypertrophy and heart failures [195].

Cancer: Since P-TEFb stimulates transcription elongation of many cellular genes, it is not surprising that P-TEFb is involved in many types of cancer [51,135,196]. An example of P-TEFb’s direct involvement in cancer is mixed-lineage leukemia (MLL), where frequent translocation and genetic rearrangement occur between the MLL gene and components of SEC, which is a potent recruiter of P-TEFb [21,145]. A majority (~50% of infant cases and ~75% of adult cases of acute lymphoblastic leukemia with MLL rearrangement) of MLL rearrangement result in in-frame fusion proteins between MLL and SEC’s AFF4 subunit [56]. MLL rearrangement also occurs with other SEC subunits, namely ENL and AF9 [144]. These MLL rearrangements lead to dysregulation of transcription elongation by P-TEFb. Another example of P-TEFb’s direct involvement in cancer is the expression and the function of c-Myc, which are upregulated in many types of cancer [6]. The expression of the c-Myc gene is regulated by P-TEFb recruited by Brd4, and removal of Brd4 from the c-Myc locus by BETi results in a potent anti-tumor effect [157,158]. Moreover, direct interaction between c-Myc and P-TEFb is required for c-Myc’s function to promote RNAPII pause-release regulated by DSIF [46,47,133,197]. Therefore, P-TEFb plays a central role in c-Myc-dependent tumorgenesis. Furthermore, P-TEFb binds to and regulates the activities of many TFs involved in cancer, which includes hormone receptors such as ER and AR [48,49,50]. Also, in various types of cancers, P-TEFb activity is aberrantly upregulated, presumably via a shift of P-TEFb equilibrium between free P-TEFb and 7SK snRNP [15,198,199,200]. In particular, mutations in LARP7 observed in various breast cancer patients cause a disruption of 7SK snRNP and subsequent constitutive activation of P-TEFb-dependent transcription [198,200]. In some types of cancer including ovarian cancer, pancreatic cancer, breast cancer, and osteosarcoma, on the other hand, P-TEFb expression (CDK9 and/or CycT1) is aberrantly upregulated [201,202,203,204,205], resulting in global stimulation of P-TEFb-dependent transcription. Although P-TEFb protein level is closely correlated with cells’ proliferative state [14,15,206] as described above, it is still unclear whether the upregulation of P-TEFb expression in these cancer cells is a cause or a consequence of high rates of cell proliferation. There are also reports suggesting that P-TEFb interacts with tumor suppressors such as P53 and RB [207,208]; this affects cellular response to DNA damage. Importantly, although global activation of transcription elongation by P-TEFb is associated with cancer, it is not completely understood whether (or which) particular sets of P-TEFb-dependent genes are responsible for tumorgenesis except for a few anti-apoptotic genes such as MCP-1 or XIAP-1 being among the genes immediately responding to P-TEFb release and activation [19,76,77]. Perhaps, different sets of P-TEFb-target genes are upregulated in different types of cancer depending on how P-TEFb function is deregulated [209,210,211]. Interestingly, due to the negative feedback self-regulatory loop described above (Figure 2), a temporary upregulation of P-TEFb activity leads to re-incorporation of P-TEFb into 7SK snRNP by newly synthesized HEXIM1 protein, which causes cell growth arrest [137]. Therefore, many anti-proliferative agents including HDACis and BETis activate P-TEFb by releasing it from 7SK snRNP activators [128,129,132,137,138,139,140,158]. Although it is largely unknown whether HEXIM1 expression is also upregulated in cancer cells with constitutive upregulation of P-TEFb expression and activity, it is fascinating to speculate that there is a specific mechanism to alleviate this negative feedback system in cancer cells.

Inflammation/autoimmune diseases: Several inflammatory cytokines and downstream TFs are upregulated in autoimmune diseases, and many of these TFs utilize P-TEFb [146,199,212,213,214,215,216] (Table 1). Namely, the Tumor Necrosis Factor alpha (TNF-α) signaling releases P-TEFb from 7SK snRNP via unknown mechanisms, and promotes direct interaction between P-TEFb and NFκB [45,131]. Also, Interleukin 6 (IL6) induces interaction between STAT3 and P-TEFb, resulting in the upregulation of STAT3-target genes, although it is currently unclear whether IL6 signaling per se releases P-TEFb from 7SK snRNP or whether STAT3 directly recruits P-TEFb without other factors such as Brd4 or SEC [63]. In addition, various TFs involved in autoimmunity bind to P-TEFb. These TFs include CIITA and AIRE [66,67]. Interestingly, transgenic mice constitutively expressing CIITA in joints via the collagen type II (CII) promoter exhibit a strong rheumatoid arthritis (RA)-like phenotype [217]. Therefore, it is likely that P-TEFb is constitutively active and involved in upregulation of transcription elongation in RA tissues, and dysregulation of P-TEFb-dependent transcription plays a critical role in RA pathogenesis. Inhibition of Cdk9 kinase activity in CII-induced arthritis model mice delays disease onset and reduces the severity of arthritis [213]. Similarly, inhibition of Cdk9 kinase reduces expression of most inflammatory mediator genes in human chondrocytes [214]. Cdk9 inhibition also prevents cartilage matrix degradation induced by a proinflammatory cytokine (IL1β) presumably through suppression of catabolic genes including matrix metalloproteinase genes [214]. Importantly, P-TEFb is a major regulator of apoptosis and the life span of neutrophils, whose abnormally long life span is associated with RA pathogenesis [218]. There are several reports showing that inhibitors of P-TEFb’s CDK9 kinase activity can be used to treat RA [219,220].

Infectious Diseases other than HIV: Since P-TEFb was first identified as a cellular co-factor of HIV transcription, many other viral proteins were found to interact with P-TEFb [221]. It is not surprising that Tat proteins from other lentiviruses such as simian immunodeficiency virus (SIV), equine infectious anemia virus (EIAV), and bovine immunodeficiency virus (BIV), and the Tax protein from human T-cell leukemia virus type 1 (HTLV-1) binds to CycT1 [31,222,223,224]. Moreover, many viral proteins interact with P-TEFb, and these interactions mediate viral replication and/or pathogenesis [221]. During Herpes Simplex Virus (HSV)-1/2 infection, for example, an immediate early gene product, ICP22, binds to Cdk9 and inhibits its kinase activity and P-TEFb-dependent viral transcription, promoting viral latency [225]. ICP22’s inhibitory effect on viral transcription is overcome by viral transactivator VP16 which also binds to P-TEFb. This could represent a mechanism of the transition between latent and productive infection of HSV [226,227]. Similarly, K-cyclin from KHSV [228], the large E1A protein from adenovirus [229], and EBNA2 from Epstein-Barr Virus (EBV) [230] interact with P-TEFb and recruit cellular transcription machineries to viral promoters. Retroviruses and many DNA viruses utilize RNAPII for viral transcription [231], and, therefore, it is conceivable that P-TEFb activity is required for the replication of these viruses. Moreover, the influenza virus RNA-dependent RNA polymerase (vRNP) also binds to P-TEFb, which facilitates the interaction between vRNP and cellular RNAPII, and stimulates viral transcription [232]. Thus, P-TEFb (CDK9) inhibitors could be promising anti-viral agents to treat various infectious diseases [221]. As such, a potent CDK9 inhibitor, FIT039, inhibits replication of multiple DNA viruses [233,234].

## 8. Approaches to Manipulate P-TEFb Functions

Since P-TEFb is a master regulator of transcription elongation, its deregulation is involved in many diseases. P-TEFb is an excellent target for the development of therapeutic approaches to treat these diseases. However, P-TEFb is controlled by multiple complex regulatory mechanisms as described above, and each P-TEFb-regulatory mechanism can be targeted alone or in combination depending on how P-TEFb is deregulated in each disease. Approaches to manipulate P-TEFb include inhibition of CDK9 kinase activity, activating CDK9 kinase activity, shifting P-TEFb equilibrium, changing P-TEFb protein levels, and modulating the interaction between P-TEFb and its recruitment factors.

## 9. Suppressing P-TEFb Activity

CDK9 kinase inhibitors: Since P-TEFb functions via the kinase activity of its CDK9 subunit, the most common approach to manipulate P-TEFb is to inhibit CDK9′s kinase activity. One of the first examples of potent and specific CDK9 inhibitors was Flavopiridol (Alvocidib) [164,235]. Flavopiridol was originally developed as an anti-tumor drug with a potent inhibitory effect on CDK4 and CDK6 [236,237]. However, a later study revealed that Flavopiridol inhibits CDK9 more potently (IC50 for CDK9 is 4–6 nM whereas IC50 for CDKs4/6 is more than 20nM) [164] (Table 2). Flavopiridol was first examined for its inhibitory effect on HIV transcription and replication, which was the most obvious system where P-TEFb is directly involved, and exhibited a very potent and specific anti-HIV effect [164]. Since then, Flavopiridol has been considered as a golden standard for development and evaluation of new CDK9 inhibitors [238] [239,240]. Flavopiridol is currently undergoing a clinical trial as a treatment for acute myeloid leukemia (AML) [241], but it is also effective in arthritis and atherosclerotic plaque formation [242,243]. Because of the high potential of CDK9 inhibitors as therapeutic agents for various diseases including HIV/AIDS and cancer, many new CDK9 inhibitors have been developed (Table 2). Major pharmaceutical companies such as Bayer (BAY1143572/ Atuveciclib), Pfizer (PHA-767491), Eli Lilly (LY2857785), and AstraZeneca (AZ5576) have developed their own CDK9 inhibitors which are being tested in pre-clinical and clinical studies of many disease models including B- or T-Cell Leukemia/Lymphoma, esophageal adenocarcinoma, acute myelogenous leukemia, primary peritoneal carcinoma, chronic lymphocytic leukemia, relapsed multiple myeloma, non-Hodgkin’s lymphoma, acute lymphoblastic leukemia, acute biphenotypic leukemias, advanced breast cancer, non-small cell lung cancer, solid advanced tumors, etc. [244,245,246,247,248,249]. Including Alvocidib, there are several CDK9 inhibitors including Dinaciclib, Seliciclib, Atuveciclib, Voruciclib, and SNS-032, which are being examined in clinical trials for treatments of these diseases [250,251,252,253] (Table 3). Moreover, many new and potent CDK9 inhibitors such as P276-00, CDKI-73, i-CDK9, Wogonin, CCT068127, MC180295, ABC1183, FIT-039, PC585, NVP-2, etc., have been reported and tested [220,234,254,255,256,257,258,259,260,261] (summarized in Table 2).

Increasing evidence indicates that inhibiting CDK9 kinase activity is a promising approach for chemotherapy in many types of cancer [74]. However, since these CDK9 kinase inhibitors target the catalytic domain of CDK9, which shares common structural motifs with other CDKs, most CDK9 inhibitors have broad specificities of target kinases (Table 2). For example, Flavopiridol inhibits CDK4/6 with slightly higher (5–6 fold) IC50 than CDK9, and Dinaciclib is also known as a potent inhibitor of CDK1 and CDK12 [74]. In addition, their inhibitory effects on “new” transcriptional Cyc/CDK complexes (CDK11/CycL, for example) have not been tested. Therefore, it is still questionable whether these CDK9 inhibitors elicit anti-proliferative effects via merely inhibiting CDK9 or inhibiting multiple CDKs. Furthermore, Sheltzer and colleagues recently tested the actual target of several compounds under clinical trials for cancer therapies by measuring their anti-proliferative effects in cells where their original targets were knocked out by CRISPR/Cas9 screening, and discovered that many compounds elicit their anti-proliferative activity via off-target effects, not via blocking pathways they were originally designed for [264]. We also demonstrated previously that many commercially available PKC inhibitors inhibit CDK9 kinase activity with IC50 values similar to that for PKC [265]. Interestingly, Grana and colleagues recently demonstrated that distinct sets of genes are affected by different strategies of blocking CDK9 (pharmacological inhibition of CDK9, blocking CDK9 by dominant negative or kinase-negative CDK9 and/or CDK9 knockdown by siRNA) [209,210]. In addition, recent in vitro and in vivo studies indicate that other than RNAPII CTD and N-TEFs, many cellular proteins involved in various biological pathways including signal transduction, transcription, mRNA processing, etc., can be substrates of CDK9 kinases [113]. These results indicate that CDK9 inhibitors can elicit very broad effects on cells, depending on compounds, conditions, cell types, etc., and it is necessary to carefully evaluate these effects to reduce unwanted side effects. Thus, the anti-proliferative effects of most CDK9 inhibitors are likely to be via their inhibitory effects on multiple protein kinases.

Another important issue to consider for using CDK9 inhibitors for therapies is the fact that inhibiting CDK9/global transcription elongation per se promotes P-TEFb release and activation as a cellular response to stress [15,126]. This phenomenon was originally observed by Bensaude and colleague, eventually leading to the discovery of 7SK snRNP and the unique self-regulatory system of P-TEFb [266] (Figure 2). This suggests that inhibiting CDK9 in its “active pool” will result in temporary upregulation of transcription by P-TEFb extracted from its inactive pool. This could also lead to HEXIM1 expression and 7SK snRNP reformation [136,137]. Because of this complex multilayered P-TEFb regulation, it is difficult to assess the outcome of CDK9 inhibitor treatment for therapeutic purposes.

**Figure 4:**
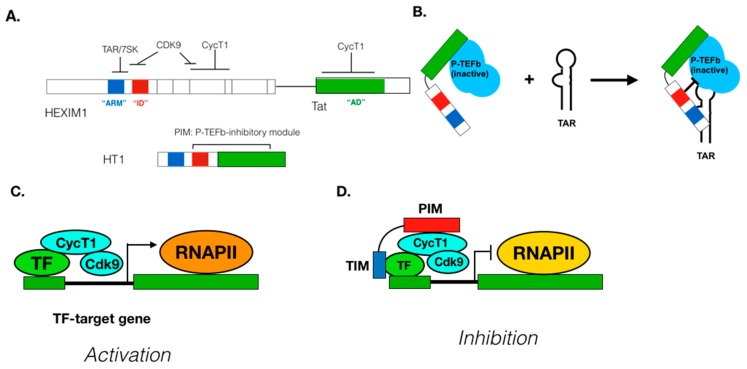
(**A**) Structure of the HEXIM1-Tat fusion peptides used. Functional domains used from HEXIM1 and Tat include a HEXIM1 Arginine-Rich Motif (ARM, blue box, residues 150–162) that binds to HIV TAR RNA, a HEXIM1 inhibitory domain (ID, red box, residues 200–211) that inhibits CDK9, and a Tat transactivation domain (AD, green box, residues 1–48) that binds to P-TEFb. ID and AD form a potent P-TEFb inhibitory motif (PIM). HT1 was constructed by fusing ARM and ID from HEXIM1 and AD from Tat. (**B**) A model for HT1’s inhibitory mechanism. HT1 recruits P-TEFb via AD to HIV’s Transactivation Response (TAR) element through the interaction between HT1’s ARM and TAR. HT1’s ID inhibits Cdk9’s kinase activity in the HT1/P-TEFb/TAR complex, blocking HIV transcriptional elongation. (**C**) Transcription factor (TF)-specific recruitment of P-TEFb to its target genes. Various DNA-bound and unbound TFs (green oval) directly interact with P-TEFb to recruit it to their target gene. P-TEFb then stimulates transcriptional elongation by phosphorylating RNA Polymerase II (RNAPII, orange oval). Such TFs include cMyc, NFκB, STAT3, MyoD, etc. (**D**) A strategy of creating TSPis, which contain a PIM (red) that inhibits P-TEFb (light blue ovals) and a TF interaction module (TIM, blue) that binds to specific TFs. In cells, these TSPis interact with their target TF and inhibit transcriptional elongation in a TF-specific manner.

Target gene/TF-specific P-TEFb inhibitors: Since P-TEFb regulates transcription of most cellular genes, pan-CDK9 inhibitors block expression of many disease-related and unrelated genes. Therefore, the usage of pan-CDK9 inhibitors for chemotherapies requires extra caution to avoid severe side effects. CDK9 inhibitors highly specific for its target genes would be highly beneficial for the development of a safe and effective treatment for diseases where P-TEFb-dependent transcription is involved although there are no such P-TEFb inhibitors available so far. P-TEFb can be recruited to its target genes by gene-specific and non-specific mechanisms (Figure 3), which is a critical step for the determination of P-TEFb’s target specificity. Compounds targeting this step would exhibit selective inhibition of specific genes. For example, a recent report by Shilatifard and colleagues demonstrated that small compounds KL1 and KL2 that disrupt the interaction between P-TEFb and the AFF subunit of SEC exhibit potent anti-proliferative activities against Myc-induced cancers [267]. Moreover, several DNA-bound and unbound TFs directly interact with P-TEFb, which could be targeted for designing/screening inhibitors that disrupt the interaction between P-TEFb and TFs (Figure 4). To this end, we previously designed a peptide chimera (HT1) that is highly specific for the interaction between P-TEFb and HIV Tat protein [268] (Figure 4A). HT1 contains a P-TEFb-interacting motif (PIM), which binds to and inhibits P-TEFb, and another peptide motif that specifically binds to HIV TAR RNA and exhibits a potent and specific inhibition of HIV transcription and viral reactivation from latently infected cells [268] (Figure 4B). Taking a similar approach, TF-specific P-TEFb inhibitors (TSPis) can be created by combining PIM and a peptide motif targeting specific TFs (Figs.4C to D). Such inhibitors will be highly effective for treatment of various diseases, including autoimmune/inflammation diseases and/or cancer.

PROTAC: Proteolysis Targeting Chimera (PROTAC) is a novel concept of small compounds that can degrade their target proteins in cells via the proteasome degradation pathway [269]. PROTAC consists of two active components inter-connected by a linker [269]. One component directly interacts with its target (i.e., CDK9), while the other, the thalidomide derivative pomalidomide, recruits cereblon (CRBN) E3 ubiquitin ligase and mediates the target protein’s degradation. The first PROTAC anti-cancer candidates have now entered clinical trials [270]. For PROTAC targeting P-TEFb, various CDK9 inhibitors including Wogonin and SNS-032 were chosen [260,271,272]. However, PROTAC for CDK9 possesses the same problem, in terms of target specificity, as other classical CDK9 kinase inhibitors. Non-promiscuous CDK9 inhibitors must be used for PROTAC to prevent degradation of protein kinases other than CDK9. Also, since P-TEFb is essential for cells, constant degradation of CDK9 by a long-term PROTAC treatment would cause severe toxicity to normal cells although intermittent short-term treatment might be sufficient to elicit long-term transcriptional suppression of CDK9′s target genes [273].

## 10. Increasing P-TEFb Activity

Since P-TEFb activity is regulated in cells via a tight equilibrium between “free” active P-TEFb (associated with TFs, SEC, Brd4, and other P-TEFb-recruiters) and inactive P-TEFb in 7SK snRNP (Figure 2), compounds that can change this equilibrium can act as effective modulators for P-TEFb activity [15,126]. Compounds that can release P-TEFb from its inhibitory 7SK snRNP complex and thus stimulate CDK9 kinase activity have been particularly tested as anti-HIV/AIDS therapies [161,162]. To eradicate HIV and cure AIDS, it is necessary to eliminate the reservoir of latently infected cells scattered throughout the body [174,178]. A shock and kill approach, where the virus is flushed out of this reservoir, while new rounds of infection are blocked by the best possible cART, represents a potential component of HIV anti-latency therapy [171]. To optimally reactivate HIV from latently infected cells, it is necessary to stimulate HIV transcription where P-TEFb activity is essential [156,161,171]. There are a number of “latency reversal agents” (LRAs) currently being tested for this approach. In particular, HDACis and BETis are among the most potent/promising ones [172,178,274]. Previously we demonstrated that most LRAs efficiently release P-TEFb from 7SK snRNP and stimulate CDK9 activity [128]. Other compounds such as HMBA; PKC agonists such as PMA, ingenols, and Bryostatin-1; DNA-damaging agents; and nucleotide analogues including Azacytidine also release P-TEFb [161,162,171,172,178,275]. Recently, we established an assay to monitor P-TEFb-release in live cells based on bimolecular fluorescence complementation (BiFC) [275]. This Visualization of P-TEFb Activation in Cells (V-PAC) assay quickly tests the ability of compounds of interest to activate P-TEFb by releasing it from 7SK snRNP [275]. For “shock and kill” approaches for the treatment of HIV-infected patients, it is also necessary to increase the expression levels of CycT1 prior to the release of P-TEFb from 7SK snRNP, since CycT1 protein levels are reduced to undetectable levels in resting CD4+ T cells by post-transcriptional mechanisms [185,186,187]. Although the precise mechanism of this CycT1 protein down-regulation is yet to be fully understood, various compounds such as PKC agonists PMA, Bryostatin-1, and ingenols increase CycT1 protein levels [155,161,162]. Therefore, a combination of a P-TEFb-releaser and a PKC agonist elicits a potent induction of HIV transcription in latently infected cells [161,171,274]. Interestingly, extracts from *Euphorbia kansui,* a plant used in Chinese traditional medicine for treatment of fluid retention, cancer, or ascites, contains a high concentration of various ingenol derivatives, and exhibits potent HIV reactivation in combination with HDACis or BETis [276].

P-TEFb-releasers/activators are also effective as anti-cancer agents. Because of the P-TEFb self-regulatory negative feedback mechanisms described above (Figure 2), P-TEFb release and activation immediately results in HEXIM1 expression and subsequent re-formation of 7SK snRNP and cell growth arrest [136]. Therefore, a common immediate cellular response to many anti-cancer drugs including HDACis is to release P-TEFb and activate CDK9 kinase [128,129,137]. In particular, we have demonstrated that a dihydroorotate dehydrogenase inhibitor A771726/Teriflunomide exhibits a strong anti-proliferative effect on melanoma by activating P-TEFb by its release from 7SK snRNP and expressing HEXIM1 [137].

Although many compounds from different categories (HDACis, BETis, nucleotide analogues, DNA damage agents, etc.) can release P-TEFb from 7SK snRNP, the precise molecular mechanism by which each compound releases P-TEFb requires thorough investigation. None of these compounds seem to disrupt the physical interaction between P-TEFb and 7SK snRNA or HEXIM1 directly, although such compounds have high therapeutic potential. Instead, various different upstream signaling cascades are involved in P-TEFb release by different stimuli and stresses. For example, HMBA induces the PI3K/Akt pathway, leading to P TEFb-release [130]. Also, PKC disrupts 7SK snRNP by phosphorylating HEXIM1 [265]. Phosphorylation of S175 in CDK9 also seems to be involved in this process [85]. Various different phosphatases control P-TEFb activities although their substrates, and the sites of phosphorylation affected by these phosphatases are largely unknown [80,84,86,277,278,279,280,281,282,283,284]. Defining the precise pathway and the molecular mechanism involved in the control of P-TEFb equilibrium responding to cellular stresses and stimuli is a critical step to design/develop effective agents that can modulate P-TEFb activity.

## 11. Potential Problems/Side Effects

P-TEFb regulates transcription of many genes involved in various human diseases and conditions, and, therefore, P-TEFb is an excellent therapeutic target. To this end, many CDK9 inhibitors have been developed and some of them are being tested in clinical trials [74]. However, because of these inhibitors’ broad range of activity on target kinases, it is difficult to determine whether their anti-proliferative effects are primarily due to CDK9 inhibition. In addition, P-TEFb stimulates elongation of many cellular genes which are not involved in diseases [14]. Particularly, genes immediately responding to P-TEFb activation include both anti-proliferative and anti-apoptotic genes [51,136,137,196]. Therefore, global inhibition or activation of P-TEFb might result in complex cellular responses. Both CDK9 inhibitors and CDK9 activators (P-TEFb releasers) can act as anti-proliferative agents [51,128,129,136,137,138,156,158,196]. For treatment of HIV, for example, although CDK9 inhibitors can completely block HIV replication in vitro, none of the CDK9 inhibitors are approved for treatment of HIV-infected patients mainly due to their toxicity. Therefore, special caution is required to use pan-CDK9 inhibitors, and therapeutic regimens should be carefully determined based on diseases, types of cells, target genes to inhibit, etc.

## 12. Perspectives and Future Directions

P-TEFb was first identified as an essential co-factor for HIV transcription and became a main therapeutic target for anti-HIV treatment, which turned out to be rather futile because of the high toxicity of CDK9 inhibition. Instead, discoveries regarding the involvement of P-TEFb in other diseases pushed the P-TEFb to center stage again as a potential therapeutic target. Now, more and more CDK9 inhibitors are being developed and tested in various disease models. There has been a great improvement in the target specificity of CDK9 inhibitors. In the meantime, P-TEFb releaser/activators are also being tested for treatment of HIV/AIDS as well as cancer. In the next decade, we expect to obtain highly specific and potent pan-CDK9 inhibitors. However, to achieve safe and effective therapies which target P-TEFb to treat different diseases, it is critical to obtain a thorough understanding of P-TEFb-mediated transcription. There are many unanswered questions regarding P-TEFb including the following. *How does P-TEFb affect its target genes via different transcriptional cues? Why are P-TEFb target genes aberrantly regulated in each disease model? Which molecular pathways are involved in P-TEFb release from 7SK snRNP by different stresses and stimuli? How are the protein levels of CycT1 (and CDK9) regulated by cellular states? What are the exact roles of P-TEFb-binding proteins?* Answering these questions will help to achieve the ultimate goal of establishing therapeutic methods to manipulate P-TEFb activity on specific target genes or pathways involved in different diseases and conditions.

## Figures and Tables

**Table 1:** Cellular transcription factors that interact with P-TEFb.

Transcription Factor	Category *	Refs
NFκB	DNA-binding transcription activator activity	[45]
Myoblast Determination Protein 1 (MyoD)	DNA-binding transcription activator activity	[62]
Estrogen Receptor	nuclear receptor activity	[49]
Androgen Receptor	nuclear receptor activity	[48]
Signal Transducer and Activator of Transcription 3 (STAT3)	DNA-binding transcription factor activity	[63]
cMyc	DNA-binding transcription activator activity	[47,64]
Myocyte Enhancer Factor-2 (MEF2)	DNA-binding transcription activator activity	[65]
Class II Transactivator (CIITA)	activating transcription factor binding	[66]
Autoimmune regulator (AIRE)	DNA-binding transcription activator activity	[67]
*SRY*-related HMG-box 2 and 10 (Sox2/Sox10)	DNA-binding transcription factor	[68]
IKAROS	DNA-binding transcription factor activity	[69]
Peroxisome Proliferator Activated Receptor gamma (PPARγ)	DNA-binding transcription factor activity	[70]
Lim Domain Binding 1 (Ldb1)	enhancer sequence-specific DNA binding	[71]

* www.uniprot.org.

**Table 2:** Examples of Cdk9 inhibitors.

Inhibitor	Alternative Names				IC50	(nM)					Refs
		Cdk1	cdk2	cdk3	cdk4	cdk5	cdk6	cdk7	cdk9	Other targets	
Flavopiridol	Alvocidib	30–400	100	410	20–40	110	60	110–300	6		**
BAY1143572	Atuveciclib	1093	997	893	n.d.	n.d.	n.d.	n.i.	6	GSK3	**
PHA-767491	CAY10572	250	240	n.d.	n.d.	460	n.d.	n.i.	34	cdc7	**
LY2857785		n.d.	n.d.	n.d.	n.d.	n.d.	n.d.	246	11		**
Dinaciclib	SCH727965	3 to 10	1	1	n.d.	0.8–1	n.d.	5	1 to 4		**
Roscovitine	Seliciclib	330–650	170–700	1500	n.i.	280	5100	800	230		**
Voruciclib		5.4–9.1	n.d.	n.d.	3.9	n.d.	2.9	n.d.	0.6–1.6		[252]
SNS-032		480	38–48	56	925	740	n.d.	62	4		**
P276-00	Riviciclib	79	224–2500	n.d.	63	n.d.	n.d.	2870	40		**
CDKI-73		4 to 8	30–33	n.d.	8	n.d.	38	91–134	4 to 6		**
i-CDK9	HY16462, CDK9-IN-2	1700	240	n.d.	1800	n.d.		2	0.4		**
Wogonin	Vogonin	n.d.	n.d.	n.d.	n.d.	n.d.	n.d.	12300	190		**
TG02	Zotiraciclib	9	5	8	n.d.	n.d.	n.d.	4	3	Lck, Fyn, JAK, FLT3, etc.	[262]
FIT-039			n.i.	n.d.	30000	n.i.	n.i.	n.i.	5.8		[234]
CCT068127		1100	10–110	n.d.	4800	70	6200	520	90		[258]
AZD4573		37	n.i.	n.d.	1100		1100	1100	14		[263]
MC180295		138	233–367	399	112	186	712	555	5		[261]

** http://www.rustreg.upol.cz/CDKiDB/. n.d.: not determined. n.i.: no inhibition.

**Table 3:** Cdk9 inhibitors enrolled in clinical trials. (https://clinicaltrials.gov).

Inhibitor	Alternative Names	Conditions or Diseases	Phase
Flavopiridol	Alvocidib	B-cell Chronic Lymphocytic Leukemia	2
		Prostate cancer	2
		(26 other phase 2 clinical trials and 33 phase 1 clinical trials)	
BAY1143572	Atuveciclib	Advanced Cancer	1
		Acute Leukemia	1
Dinaciclib	SCH727965	Refractory Chronic Lymphocytic Leukemia	3
		Stage IV Melanoma	2
		(Three other phase 2 clinical trials and 11 phase 1 clinical trials)	2
Roscovitine	Seliciclib	Cushing Disease	2
		Moderately to Severely Active Ulcerative Colitis	2
Voruciclib		B-cell malignancies or AML	1
SNS-032		Advanced B-lymphoid Malignancies	1
		Advanced solid tumor	1
P276-00	Riviciclib	Head and Neck Cancer	2
		Melanoma	2
		(Four other phase 2 clinical trials and four phase 1 clinical trials)	
TG02	Zotiraciclib	Brain Timor	2
		Advanced Hematological Malignancies	1
		(Three other phase 1 clinical trials)	
AZD4573		Relapsed or Refractory Hematological Malignancies	1
TP-1287		Advanced Solid Tumor	1
